# Prevalence of Postpartum Depression and Associated Risk Factors Among Women in Jeddah, Western Saudi Arabia

**DOI:** 10.7759/cureus.14603

**Published:** 2021-04-21

**Authors:** Najma A Alsayed, Jamelah F Altayyeb, Laura S Althuniyyan, Shatha K Alzubaidi, Fayssal Farahat

**Affiliations:** 1 College of Medicine, King Saud bin Abdulaziz University for Health Sciences, Jeddah, SAU; 2 Medicine, King Abdullah International Medical Research Center, Jeddah, SAU; 3 Medicine, ​King Abdullah International Medical Research Center, Jeddah, SAU; 4 Infection Prevention and Control, King Abdulaziz Medical City, Jeddah, SAU

**Keywords:** postpartum depression, postpartum, depression, jeddah, saudi arabia, prevalence, risk factors

## Abstract

Background

Postpartum depression (PPD) is a significant psychological disorder that can affect women during or after pregnancy. Its risk increases throughout the first 90 days and may last up to almost two years, which can create an overall burden on society. Although the etiology is unknown yet, there are risk factors that contribute to developing PPD. This study aims to assess the prevalence of PPD and the risk factors among women in Jeddah, western Saudi Arabia in 2019.

Methods

A cross-sectional study was conducted in infant vaccination clinics of the primary healthcare centers (PHC) of the Ministry of Health (MOH) and Ministry of National Guard (MNGHA) Jeddah, Saudi Arabia. Data were collected through a self-administered questionnaire with Edinburgh Postnatal Depression Scale (EPDS) from mothers up to four months postpartum.

Results

Of the 172 postpartum women, the study estimated the prevalence of postpartum depression to be 20.9%. The significant risk factors predicted by multivariate regression analysis were history of previous depression (odds ratio {OR}=4.7; 95% confidence interval {CI} 1.9 to 11.5), difficult life event interval (OR=3.5; 95% CI 1.1 to 10.7), and attitude toward pregnancy (OR=2.1; 95% CI 0.9 to 4.5).

Conclusion

A fairly high prevalence of postpartum depression was revealed among the females in Jeddah. Therefore, we recommend screening of mothers after delivery to help early intervention and management along with psychosocial support.

## Introduction

After childbirth, women may experience some difficulties that affect them physically, socially, and psychologically. Despite their importance, psychological consequences are not usually addressed. They can vary from having baby blues - a feeling of sadness that is experienced by a woman shortly after giving birth and usually lasts less than two weeks that is typically accompanied by other symptoms such as mood swings and loss of appetite - to having postpartum depression (PPD) [1,2]. PPD is a significant psychological disorder that can affect women during and after delivery and its risk increases throughout the first 90 days after delivery and may last up to almost two years [3]. PPD is defined in the Diagnostic and Statistical Manual of Mental Disorders, fifth edition, as a major depressive episode with onset in pregnancy or within four weeks post-delivery [4]. 

PPD is fairly common and its prevalence is significantly higher in developing countries. Some studies have been done in Saudi Arabia, for example, in Dammam 17.8% prevalence of PPD has been reported in 2014, while another study in Jeddah showed a prevalence of 23.9% in 2015 [5,6]. According to studies done in the Gulf region, the prevalence of PPD was estimated to be 11.7% in Kuwait and 33% in Dubai, United Arab Emirates [7,8]. Globally, a study in the United States estimated the prevalence of PPD to be 14.9% while in Australia it has been reported to be 16.1% [9,10]. 

Unfortunately, the psychological health of mothers is usually overlooked by postnatal care, which is more focused on obstetric aspects and infant’s health. Symptoms of depression - and in severe cases - tendencies of self-harm or even harm to the baby are usually unrecognized and therefore untreated. Consequences of undiagnosed PPD do not only affect the mother and her infant but continue to disturb the family dynamic through a failure of the mother to accomplish her role as a caregiver [5]. This can create an overall burden on society. Although the etiology is unknown yet, there are risk factors that contribute to developing PPD. Stressful life events, a history of any psychiatric illness or PPD, and lack of social support are considered the most reported ones. Unplanned pregnancy, number of pregnancies, and undesired pregnancy are considered obstetric-related risk factors. Marital status and employment are sociodemographic factors that contribute to PPD. Other precursors such as mode of delivery, breastfeeding, prolonged labor, pregnancy complications, and giving birth for the first time must also be taken into consideration when assessing PPD risk factors [11]. 

In previous studies, researchers have identified several limitations. Some of these limitations are related to small sample size, use of only one measuring scale, and participants drop out or non-response [12]. Physical/nutritional problems interfering with the results like hemoglobin levels were also identified [13]. The lack of studies in Saudi Arabia about postpartum depression is considered a significant limitation. Providing data about PPD prevalence could contribute to raising awareness, help in preventing its occurrence in mothers with the acknowledged risk factors, and enhance the diagnostic processes resulting in better treatment. This study aims to estimate the prevalence and assess risk factors of PPD among women within four months in Jeddah, western Saudi Arabia in 2019. 

This article has been presented in The Third Annual Research Forum for College of Medicine held at King Saud bin Abdulaziz University of Health Sciences Jeddah, Saudi Arabia on February 18, 2021.

## Materials and methods

A cross-sectional study has been conducted in infant vaccination clinics of the primary healthcare centers (PHC) of the Ministry of Health (MOH) and Ministry of National Guard (MNGHA) Jeddah, western Saudi Arabia. There are only three MNGHA PHCs in Jeddah, and we conveniently chose the specialized polyclinic based on the clinics' number per week. The MOH PHCs were selected by simple random sampling technique, two centers out of 55 were selected to represent the northern (Ash Shati PHC) and southern (Al Jamea PHC) areas of Jeddah. The previous three centers were allocated to represent all the population of Jeddah. Based on previous literature of 17.8% prevalence of PPD, the estimated sample size was calculated and the minimum required sample size was 246 at 95% confidence interval, 5% margin of error, and 80% study power [5]. This number was increased to 300 to overcome 20% non-response. A convenient non-probability sampling technique was applied to select the required sample size among mothers attending infant vaccination clinics at primary healthcare centers. All mothers delivered within four months and willing to participate were selected. 

Data were collected using a valid Arabic version of the Edinburgh Postnatal Depression Scale (EPDS). The internal reliability of the scale was 0.84 (Cronbach's alpha) [14]. The EPDS has 10 questions, and the scoring is as the following: a score less than eight points shows that depression is unlikely, a score of nine to 11 considered depression possible, a 12-13 score interprets fairly high possibility of depression, +13 scores and higher show positive results, and the cutoff point is 13+ [14]. Additionally, a questionnaire adopted from Alasoom and Koura with permission was applied to assess risk factors and sociodemographic data of the participants [5]. The questionnaires were self-administered and a pilot study was conducted in one of the study centers randomly selected to check feasibility and internal consistency. The collected data were analyzed by using Statistical Package for the Social Sciences (SPSS) Version 24 (IBM Corp., Armonk, NY). Descriptive statistics were used to estimate the prevalence of PPD. The chi-square test was applied to assess the association of different categorical risk factors with the occurrence of PPD. Multivariate logistic analysis was applied to identify the most significant risk factors. The level of significance was at a P-value less than 0.05.

## Results

This study included 172 mothers, 118 (68.6%) attended the MNGHA PHC specialize polyclinic, 35 (20.3%) visited Ash Shati MOH PHC, and 19 (11%) attended Al Jamea MOH PHC. Their mean age was 29 ± 5 years. The prevalence of postpartum depression in Jeddah was found to be 20.9%. According to Edinburgh Postpartum Depression Scale, 36 (20.9%) of the participants had a score of 13 or more, which refers to depression; 38 (22.1%) were possibly depressed with a score between nine and 12; lastly, 98 (57%) had a score of less than nine that refers to depression less likely.

Table 1 describes the association between PPD and the sociodemographic characteristics of the study sample. Most of the participants are married, 103 (59.9%) are married to a husband working in the military sector. The majority of the participants are housewives, 139 (80.8%) and about 93 (54.1%) have a high level of education of university or above. Having enough income was reported by 157 (91.3%) of the participants. Two participants (1.2%) were divorced and three (1.7%) had their husbands married to another woman. No significant association between PPD and the sociodemographic factors was identified.

**Table 1:** Sociodemographic characteristics PPD: postpartum depression

Sociodemographic characteristics		Less likely PPD	Has PPD/possibly PPD	Total	P-value
		(n = 98)	(n = 74)	(n = 172)	
		N (%)	N (%)	N (%)	
Age	< 30	48 (49.0)	40 (54.1)	88 (51.2)	0.510
	≥ 30	50 (51.0)	34 (45.9)	84 (48.8)	
	Mean ± SD 28.98 ±4.878				
Education	Less than university	45 (45.9)	34 (45.9)	79 (45.9)	0.997
	University or above	53 (54.1)	40 (54.1)	93 (54.1)	
Occupation	Employed/student	20 (20.4)	13 (17.6)	33 (19.2)	0.639
	Housewife	78 (79.6)	61 (82.4)	139 (80.8)	
Husband occupation	Military	62 (63.3)	41 (55.4)	103 (59.9)	0.298
	Non-military	36 (36.7)	33 (44.6)	69 (40.1)	
Income	Enough	92 (93.9)	65 (87.8)	157 (91.3)	0.165
	Not enough	6 (6.1)	9 (12.2)	15 (8.7)	

Table 2 illustrates the univariate analysis of the association between PPD and the health and social-related risk factors. More than one-third of the mothers had a history of previous depression and difficult life events, both were found to be significantly associated factors of PPD (P<0.001 and P=0.007, respectively). Another significant risk factor associated with PPD was the attitude towards pregnancy (P=0.013), 44 (25.6%) of the mothers did not want the pregnancy. Regarding social support, it was assessed using a score from zero to 10, and the median was used as a cut-off point. Females who had lower scores of support from husbands and families were significantly associated with PPD (P=0.039, 0.036, respectively). However, menstrual cycle, chronic diseases, anemia, health problems during pregnancy, and depressed family members were associated with PPD but not statistically significant. The awareness of PPD was assessed by answering a direct question. Overall, 101 (58.7%) of the participants were aware of PPD (P=0.426).

**Table 2:** Health and social-related risk factors *Fisher’s exact test. PPD: postpartum depression

Health and social-related risk factors		Less likely PPD	Has PPD/possibly PPD	Total	P-value
		(n = 98)	(n = 74)	(n = 172)	
		N (%)	N (%)	N (%)	
Menstrual cycle	Irregular	22 (22.4)	13 (17.6)	35 (20.3)	0.431
	Regular	76 (77.6)	61 (82.4)	137 (79.7)	
Chronic disease	No	90 (91.8)	67 (90.5)	157 (91.3)	0.765
	Yes	8 (8.2)	7 (9.5)	15 (8.7)	
Anemia	No	81 (82.7)	59 (79.7)	140 (81.4)	0.626
	Yes	17 (17.3)	15 (20.3)	32 (18.6)	
Health problems during pregnancy	No	79 (80.6)	6 (75.7)	135 (78.5)	0.435
	Yes	19 (19.4)	18 (24.3)	37 (21.5)	
Attitude toward pregnancy	Wanted	80 (81.6)	48 (64.9)	128 (74.4)	0.013
	Don’t want/don’t know	18 (18.4)	26 (35.1)	44 (25.6)	
Difficult life events	No	79 (80.6)	46 (62.2)	125 (72.7)	0.007
	Yes	19 (19.4)	28 (37.8)	47 (27.3)	
Difficult life events interval	No	79 (80.6)	46 (62.2)	125 (72.7)	0.012
	Last year	6 (6.1)	14 (18.9)	20 (11.6)	
	> year ago	13 (13.3)	14 (18.9)	27 (15.7)	
Previously depressed	No	89 (90.8)	49 (66.2)	138 (80.2)	0.001
	Yes	9 (9.2)	25 (33.8)	34 (19.8)	
Depressed family member	No	93 (94.9)	72 (97.3)	165 (95.9)	0.7*
	Yes	5 (5.1)	2 (2.7)	7 (4.1)	
Husband support score	≤ 7	30 (30.6)	34 (45.9)	64 (37.2)	0.039
	>7	68 (69.4)	40 (54.1)	108 (62.8)	
Family support score	≤ 8	25 (25.5)	30 (40.5)	55 (32)	0.036
	>8	73 (74.5)	44 (59.5)	117 (68)	
PPD awareness	No	43 (43.9)	28 (37.8)	71 (41.3)	0.426
	Yes	55 (56.1)	46 (62.2)	101 (58.7)	

Multivariate logistic regression analysis in Table 3 demonstrates that history of previous depression (odds ratio {OR}=4.7; 95% confidence interval {CI} 1.9 to 11.5) followed by a difficult life event interval - association between the onset of stressful life events and depression - (OR=3.5; 95% CI 1.1 to 10.7) were the most significant risk factors to PPD. The attitude toward pregnancy approaches borderline significant association (OR=2.1; 95% CI 0.9 to 4.5). 

**Table 3:** Multivariate regression analysis OR: odds ratio, CI: confidence interval

Risk factors	P-value	OR	95% CI	
			Lower	upper
Attitude toward pregnancy	0.058	2.104	0.974	4.546
Difficult event interval	0.029	3.485	1.135	10.702
Previously depressed	0.001	4.689	1.906	11.533

## Discussion

This cross-sectional study in western Saudi Arabia identified a prevalence of 20.9% PPD. This study showed a higher prevalence in comparison with a cross-sectional study in the Eastern Province of Saudi Arabia [5]. However, the prevalence was lower than that of another observational study conducted in Jeddah [6]. This variation in PPD rates could be due to the differences in the study population.

In regards to the association between PPD and the studied risk factors, history of previous depression, difficult life events interval, and the attitude towards pregnancy were statistically significantly associated with PPD. Results of the recent study had shown that PPD rates were higher in women who were previously depressed or experienced stressful life events, which is consistent with that of Alasoom and Koura [5]. The death of a loved one and family conflicts are examples of stressful life events that can result in major life changes. Stressful life events have significant negative consequences for both physical and psychological well-being including postpartum psychological well-being [5,15]. 

Support from husbands or family during the pregnancy didn’t contribute to the identified depression among the studied women. This finding was discordant with that of Alasoom and Koura, who found a significant association between non-supportive husbands and PPD [5]. Our findings were consistent with that of Al-Ghamdi et al., except for the stressful life events factor since they did not find any association between this factor and PPD [16]. In regards to the association between previous depression and the risk of PPD, our findings were consistent with those of Alasoom and Koura, Almarzouki et al., and Silverman et al., who found that the risk of PPD was significantly higher for women with a history of depression than for women without a history of depression [5,6,17].

What distinguished the current study from the previous literature in Saudi Arabia is that it gathered samples from both MNGHA and MOH PHC to find whether or not being a wife of a military national guard employee influenced the PPD rates, but no association was found. However, in contrast to our findings, previous studies have found an increased risk of PPD in military wives whose spouses were deployed during pregnancy or delivery [18,19]. 

Similar to studies conducted in Saudi Arabia, the present study did not find any association between sociodemographic characteristics like income, education, occupation/husband’s occupation, and PPD [5,13]. Age and marital status, which were associated with higher PPD rates in previous studies, did not show any significance in the current study [6,13]. However, this could be due to the low number of divorced women in this study. Another difference between the current study and previous ones was that chronic diseases, health problems during pregnancy, and anemia did not increase PPD rates [13,16].

Limitations in the present study, only two women were divorced and only three had a polygamous husband, therefore, it was difficult to determine whether or not these two factors can increase the risk of PPD. Previous studies have reported an increased risk of depression and poor mental health in polygynous women compared to monogamous women in addition to reduced life and marital satisfaction, problematic family function, and the lack of husband support. Thus, the results of the current study regarding polygamy should be taken into consideration [20-22]. Another limitation to this study was the possible presence of a recall bias since our questionnaire had a number of questions that asked about past events or experiences, and the answers to these questions depended heavily on the accuracy of the mothers’ memory. That being said, the present study holds a number of advantages over previous studies conducted in Saudi Arabia by including both MNGHA and MOH PCH and assessing the military wives factor as an additional risk factor. In addition to that, the self-administered questionnaire was done in the presence of the investigators to guide the participants and to overcome participants' dropout.

The original sample size for this study was 300 mothers. However, the number had been reduced to 172 due to the coronavirus disease 2019 (COVID-19) pandemic, which limited our access to PHC, resulting in smaller sample size and unequal sample distribution between MNGHA and MOH PHC. We recommend using a larger sample size in future research in addition to distributing samples equally between centers.

## Conclusions

In conclusion, this study revealed a fairly high prevalence of postpartum depression among females in Jeddah consistent with the previous studies in other regions of Saudi Arabia. In this study, we found that history of previous depression and difficult life event intervals were the most significant risk factors. Therefore, we recommend screening of mothers after delivery to help early intervention and management along with psychosocial support. Also, we emphasize the importance of screening pregnant women during antenatal visits for early detection of any history of depression or recent difficult event in order to mitigate their impact on the incidence of depression during the postpartum period.
